# Optimizing Outcomes for Acute Mesenteric Ischemia in the Context of Hypogammaglobulinemia: A Detailed Analysis of Surgical and Medical Strategies

**DOI:** 10.7759/cureus.61531

**Published:** 2024-06-02

**Authors:** Mena Louis, Mariah Cawthon, Brian Gibson

**Affiliations:** 1 General Surgery, Northeast Georgia Medical Center Gainesville, Gainesville, USA; 2 Surgery, Northeast Georgia Medical Center Gainesville, Gainesville, USA; 3 Trauma and Acute Care Surgery, Northeast Georgia Medical Center Gainesville, Gainesville, USA

**Keywords:** acute abdominal pain, contrast allergy, immunodeficiency, mesenteric ischemia, necrotizing gangrenous inflammation, ischemic small bowel, hypogammaglobulinemia

## Abstract

Acute mesenteric ischemia is a critical condition marked by a sudden loss of blood supply to the intestines, often leading to rapid tissue necrosis and severe clinical outcomes if untreated. In the context of hypogammaglobulinemia, an immunodeficiency characterized by decreased levels of immunoglobulins, this vascular emergency becomes even more daunting. Hypogammaglobulinemia can impair the immune system's response to both infection and ischemic injury, intensifying the severity of intestinal damage. This report describes the case of a 52-year-old female with hypogammaglobulinemia who presented with severe abdominal pain. Surgical exploration revealed 100 cm of necrotic small bowel extending from 150 cm distal to the ligament of Treitz to within 10 cm of the ileocecal valve. The necrotic section was surgically removed, and primary anastomosis was performed. This instance highlights the significant impact of immunodeficiency on the progression and management of acute mesenteric ischemia, demonstrating the critical need for early intervention and tailored management strategies, especially in immunocompromised patients, to prevent severe outcomes. The case illuminates the importance of recognizing immunodeficiency as a complicating factor in acute gastrointestinal emergencies, stressing the necessity for prompt and effective medical and surgical interventions to improve prognosis and patient outcomes in complex clinical scenarios.

## Introduction

Hypogammaglobulinemia represents a pivotal challenge in clinical immunology, characterized by an abnormally low level of immunoglobulins in the blood, leading to a heightened susceptibility to infections [1]. This condition, which may arise from both congenital and acquired causes, poses significant risks to patient health due to the critical role of immunoglobulins in the immune response [2]. Primary hypogammaglobulinemia is often the result of genetic defects affecting B-cell development or function, whereas secondary forms can occur due to external factors such as medication use, chronic infections, or malignancies [1,3].

Mesenteric ischemia, a critical condition characterized by inadequate blood supply to the small intestine, presents a significant challenge in gastrointestinal medicine and surgery [4]. This ischemia can be categorized into acute and chronic forms, each with distinct etiologies and clinical presentations [4,5]. Acute mesenteric ischemia (AMI) typically arises suddenly and is most commonly caused by an embolism or thrombosis that obstructs one of the mesenteric arteries [6]. Chronic mesenteric ischemia, often termed "intestinal angina," usually develops gradually due to atherosclerotic changes in the mesenteric vessels [7,8]. Both forms require prompt diagnosis and management to prevent bowel necrosis, a complication that can lead to severe outcomes, including sepsis and death [4,5]. The complexity of diagnosing mesenteric ischemia stems from its non-specific symptoms, which often mimic other abdominal disorders, thereby requiring high clinical suspicion and rapid imaging studies for confirmation [9,10].

The association between hypogammaglobulinemia and gastrointestinal complications is well-documented, primarily focusing on an increased risk for enteric infections [11]. However, less is known about the link between severe immunodeficiency and acute gastrointestinal emergencies, such as ischemic bowel disease, which is often a life-threatening condition marked by a sudden reduction in small intestinal blood flow leading to tissue necrosis [12]. The complex interplay of impaired immunity and vascular pathology can precipitate unique clinical challenges, making the management of such conditions particularly intricate.

## Case presentation

A 52-year-old female with a history of hypogammaglobulinemia presented to the emergency department (ED) with severe abdominal pain. She reported that the pain started abruptly 12 hours before her arrival and was accompanied by non-bloody bilious emesis. Her past medical history was significant for an anaphylactic reaction to iodinated contrast, necessitating the use of CT of the abdomen and pelvis without contrast for imaging diagnostics.

**Figure 1:**
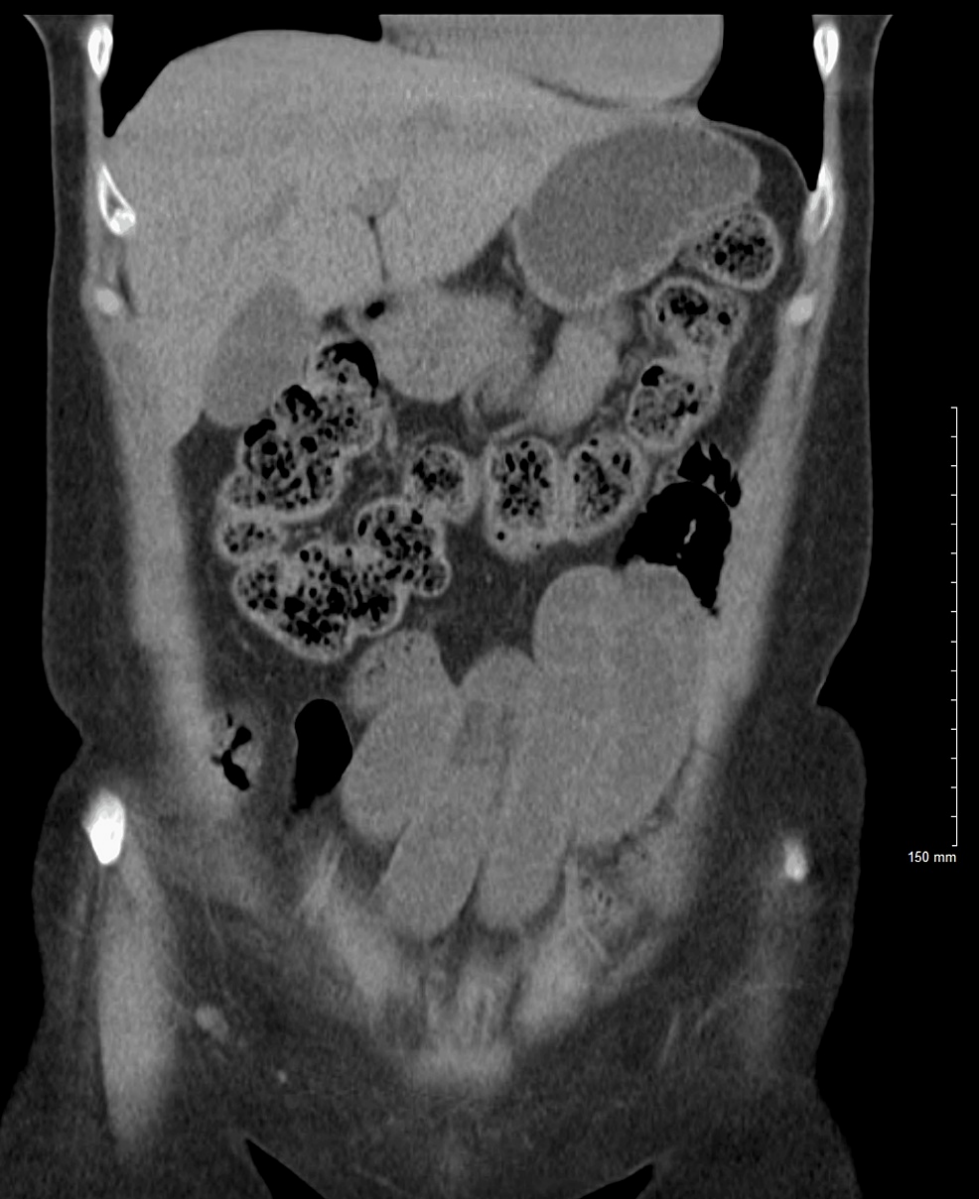
Coronal non-contrast CT of the abdomen and pelvis with no acute findings.

Upon examination, the patient exhibited signs of acute distress with tachycardia (142 bpm) and tachypnea. Her abdomen was rigid and diffusely tender with guarding and rebound tenderness, the latter indicating a severe irritation of the peritoneum typically seen in peritonitis. Initial laboratory tests showed leukocytosis and a markedly elevated lactic acid level, prompting immediate surgical intervention (Table 1). Given the clinical urgency and her contrast allergy, a non-contrast CT was performed which did not reveal any significant findings (Figure 1). A transvaginal ultrasound conducted in the ED showed a moderate amount of mildly complex pelvic free fluid, raising concerns for intra-abdominal pathology.

**Table 1:** The key laboratory results from the patient's presentation through her initial hospital course. WBC: white blood cell; Hb: hemoglobin; IgG: immunoglobulin G

Test	Result	Reference range
WBC count	18.3 K/uL	4.5-11.0 K/uL
Hb	6.1 g/dL	12.0-15.5 g/dL
Lactic acid	5.70 mmol/L	0.5-2.2 mmol/L
IgG	281 mg/dL	700-1600 mg/dL

Laboratory findings

**Figure 2:**
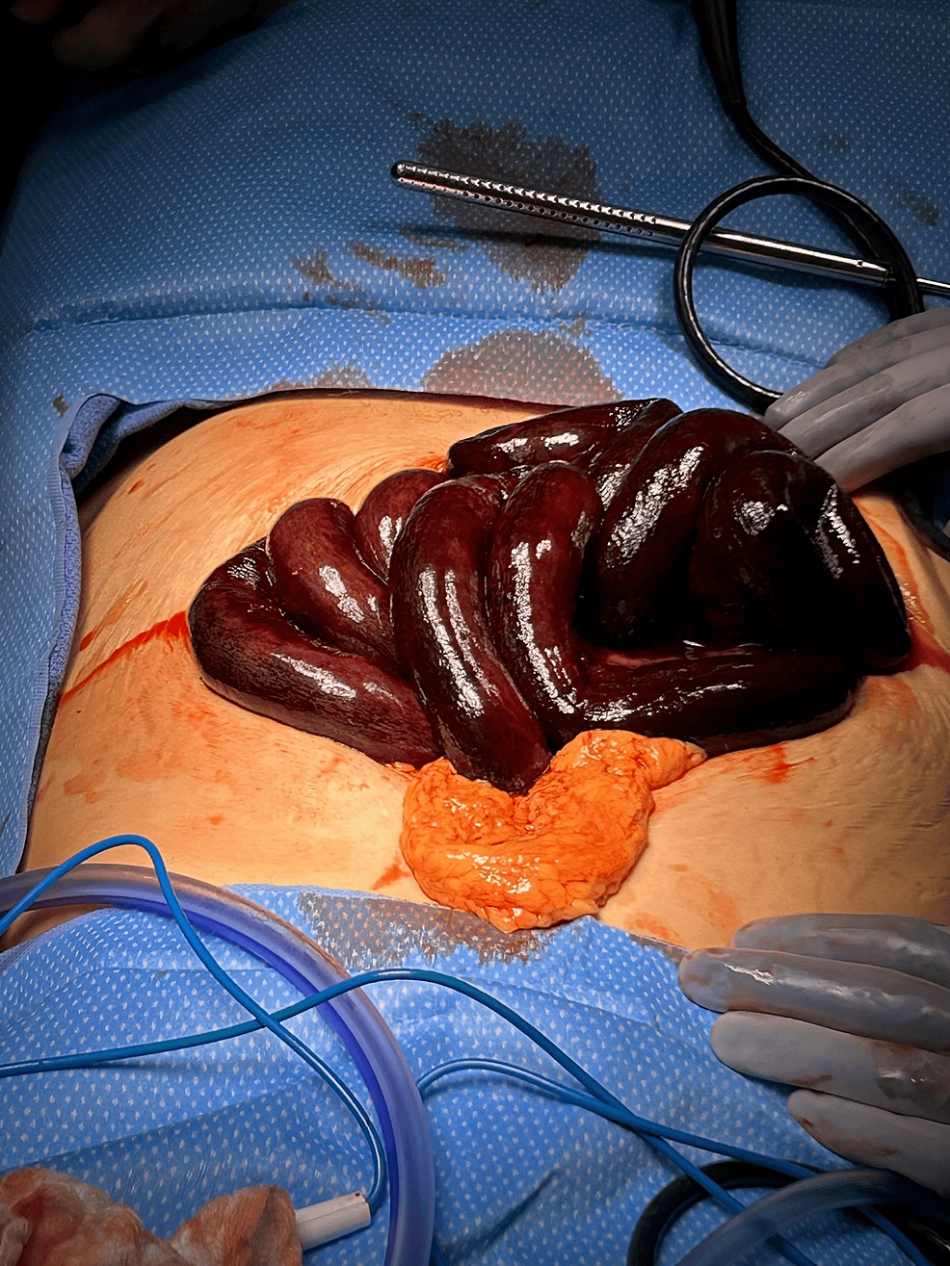
Ischemic small bowel.

Based on the clinical presentation, the decision was made to proceed with a diagnostic laparoscopy, which was quickly converted to an open procedure due to the findings. Upon entering the abdomen, 200 cc of blood was evacuated, and 100 cm of necrotic small bowel was identified and resected (Figure 2), starting 150 cm from the ligament of Treitz and extending to 10 cm from the ileocecal valve. The abdomen was thoroughly irrigated, and the decision was made to leave the bowel in discontinuity. An ABThera wound VAC was used to temporarily close the abdomen to allow for re-exploration. The second-look surgery was planned to ensure bowel viability before performing an anastomosis.

Postoperatively, the patient was maintained on intravenous antibiotics (cefepime, metronidazole, and vancomycin) and started on a heparin drip, later transitioning to warfarin. A second surgery after 24 hours confirmed the viability of the remaining bowel, and the abdomen was definitively closed. Despite the initial challenges, her postoperative recovery was uneventful, and she was discharged on a regular diet three days after abdominal closure, with ongoing anticoagulation management using warfarin and Lovenox.

## Discussion

The case of a 52-year-old female with hypogammaglobulinemia presenting with necrotic small bowel due to a vascular event underlines the critical intersection of immunodeficiency and vascular pathology in clinical practice.

The discovery of 100 cm of necrotic small bowel starting 150 cm from the ligament of Treitz and continuing close to the ileocecal valve illustrates a profound vascular compromise likely due to AMI. Such extensive bowel necrosis points towards a severe disruption in blood supply, typically via arterial occlusion, which may be either thrombotic or embolic in nature [6,12]. The severe ischemic changes coupled with necrotizing gangrenous inflammation identified in the pathology report highlight the rapid progression typical of arterial blockages.

The patient's underlying hypogammaglobulinemia adds a layer of complexity to her clinical presentation. Normally tasked with combating infections and regulating immune responses, adequate immunoglobulin levels are crucial. In their absence, not only does the risk of infection increase, but the body's ability to modulate inflammation and respond to tissue injury is also compromised [11,12]. This could potentially exacerbate the extent and severity of ischemic damage, leading to more widespread necrosis as observed in this case [3,5,12]. Postoperatively, the patient's immunoglobulin levels were checked, revealing an immunoglobulin G (IgG) level of 281 mg/dL, but she did not receive immunoglobulin therapy during her hospitalization. Instead, she was managed with an intravenous heparin infusion and was discharged home on warfarin.

Interestingly, despite the severe presentation of ischemic bowel, the patient's recovery was relatively uncomplicated. This could be attributed to the prompt surgical intervention and meticulous postoperative care, which effectively managed the immediate threats posed by the ischemic event. Additionally, her hypogammaglobulinemia did not appear to significantly impact her recovery, possibly due to the aggressive use of broad-spectrum antibiotics and vigilant monitoring for potential infections. The standard surgical treatment for ischemic bowel was adequate in this case, suggesting that while hypogammaglobulinemia increases susceptibility to infections, it may not always adversely affect the surgical outcomes if managed appropriately with targeted prophylactic measures.

The patient's allergy to iodinated contrast posed significant diagnostic challenges, complicating the ability to swiftly and accurately diagnose the extent of ischemia using standard imaging techniques. This situation emphasizes the need for alternative diagnostic strategies in patients with contrast allergies. The surgical approach-prompt laparoscopy followed by resection of the necrotic bowel highlights the necessity for rapid intervention in suspected cases of mesenteric ischemia.

The minimal inflammatory changes at the resection margins were a favorable finding, suggesting that the surgical intervention successfully removed the most severely affected bowel segments before the ischemia could progress further. This outcome highlights the effectiveness of the surgical team's decision-making and the importance of timely surgical intervention in managing AMI. In this case, the decision to convert to an open surgical approach rather than laparoscopic surgery was crucial, as it allowed for a thorough exploration and swift resection of necrotic tissue, which is supported by literature indicating that open surgery can be more effective in emergent cases of extensive bowel ischemia [13]. Timely open intervention is often preferred in such scenarios to minimize delay and ensure comprehensive assessment and treatment of the ischemic bowel.

The patient did not experience any postoperative complications such as surgical site infections (SSI), which are commonly associated with increased morbidity, extended hospital stay, and other negative outcomes [14]. This lack of complications may be attributed to the rigorous use of prophylactic antibiotics and meticulous postoperative care, which are critical in preventing SSI and ensuring a smooth recovery.

## Conclusions

This article illustrated a critical instance of ischemic small bowel with necrotizing gangrenous inflammation in a 52-year-old female with hypogammaglobulinemia, emphasizing the profound impact of vascular and immune system interplay in clinical outcomes. The significant necrosis of the small bowel, likely due to AMI caused by arterial occlusion, highlights the severity and rapid progression of ischemic conditions, especially in patients with underlying immunodeficiencies. The successful surgical intervention, which involved the resection of necrotic bowel segments and subsequent management, demonstrated the importance of prompt and decisive surgical action in the face of complex clinical presentations. While the patient's hypogammaglobulinemia and AMI coexisted, the clinical presentation, treatment, and postoperative course appeared standard for ischemic bowel disease. The hypogammaglobulinemia did not directly complicate the surgical intervention or recovery process.

## References

[1] Athni TS, Barmettler S (2023). Hypogammaglobulinemia, late-onset neutropenia, and infections following rituximab. Ann Allergy Asthma Immunol.

[2] Viallard JF (2023). Management of hypogammaglobulinemia [Article in French]. Rev Med Interne.

[3] Noto A, Cassin R, Mattiello V, Bortolotti M, Reda G, Barcellini W (2023). Should treatment of hypogammaglobulinemia with immunoglobulin replacement therapy (IgRT) become standard of care in patients with chronic lymphocytic leukemia?. Front Immunol.

[4] Navas-Campo R, Moreno-Caballero L, Ezponda Casajús A, Muñoz DI (2020). Acute mesenteric ischemia: a review of the main imaging techniques and signs. Radiologia (Engl Ed).

[5] Kärkkäinen JM (2021). Acute mesenteric ischemia: a challenge for the acute care surgeon. Scand J Surg.

[6] Bala M, Catena F, Kashuk J (2022). Acute mesenteric ischemia: updated guidelines of the World Society of Emergency Surgery. World J Emerg Surg.

[7] Gnanapandithan K, Feuerstadt P (2020). Review article: mesenteric ischemia. Curr Gastroenterol Rep.

[8] Sardar P, White CJ (2021). Chronic mesenteric ischemia: diagnosis and management. Prog Cardiovasc Dis.

[9] Olson KA, Teixeira PG (2021). Mesenteric ischemia: when and how to revascularize. Adv Surg.

[10] Selva Armadans I, Ferrés Llach J, Serra Soler S, Marcos Neira P, Ricart Martí P (2023). Mesenteric ischemia. Med Clin (Barc).

[11] Desai L, Kurien RT, Simon EG, Dutta AK, Joseph AJ, Chowdhury SD (2014). Hypogammaglobulinemia-associated gastrointestinal disease--a case series. Indian J Gastroenterol.

[12] Agarwal S, Cunningham-Rundles C (2019). Gastrointestinal manifestations and complications of primary immunodeficiency disorders. Immunol Allergy Clin North Am.

[13] Renner P, Kienle K, Dahlke MH (2011). Intestinal ischemia: current treatment concepts. Langenbecks Arch Surg.

[14] Panos G, Mulita F, Akinosoglou K (2021). Risk of surgical site infections after colorectal surgery and the most frequent pathogens isolated: a prospective single-centre observational study. Med Glas (Zenica).

